# Supplementary material for: Economic barriers to diagnostic equity: A multi-country analysis of patient costs for rapid SARS-CoV-2 testing in sub-Saharan Africa
Source: PLoS One. 2026 Jun 2;21(6):e0350288. doi: 10.1371/journal.pone.0350288 (PMC13229357; doi:10.1371/journal.pone.0350288)
Supplement: S1 File — (DOCX) [file pone.0350288.s001.docx]

**This interview is for clients exiting covid-19 testing services.**

*I will ask you a few questions about the visit you made to the testing site today. This should take less than 30 minutes to complete. Please feel free to give me your honest responses. We are not asking for your name or any information that can be used to identify you. It will therefore not be possible for anyone to link your responses to you. Some of the questions will ask about background information about yourself. Others will ask which services you accessed, how much time you have spent at the testing site and any costs you have incurred as a result. You do not need to answer any question that makes you feel uncomfortable or that you do not want to answer.*

| **1. GENERAL INFORMATION** | | |
| --- | --- | --- |
| **1.1** | Data collection date | **\| d \| d \| \| m \| m \| m \| \| y \| y \| y \| y \|** |
| **1.2** | Interviewer code | **______________________________________________** |
| **1.3** | Start time | **\| h \| h \|:\| m \| m \| *[Based on a 24 hour clock eg.15:30]*** |

| **2. DEMOGRAPHIC INFORMATION** | | |
| --- | --- | --- |
| **2.1** | Do you know your date of birth? ***[If No, skip to q.2.3]*** | ⬜ Yes ⬜ No |
| **2.2** | Date of Birth ***[k.m. 27 SEP 1957]*** | **\| d \| d \| \| m \| m \| m \| \| y \| y \| y \| y \|** |
| **2.3** | What is your approximate year of birth | **\| y \| y \| y \| y \|** |
| **2.4** | Which of the following best describes you? | ⬜ Male ⬜ Female ⬜ Other ***[indicate appropriately]*** |
| **2.5** | What is the highest level of education you have attended (Malawi) | ⬜ No education or pre-school/nursery school only  ⬜ Adult literacy education  ⬜ Standard 1  ⬜ Standard 2  ⬜ Standard 3  ⬜ Standard 4  ⬜ Standard 5  ⬜ Standard 6  ⬜ Standard 7  ⬜ Standard 8  ⬜ Form 1 of secondary  ⬜ Form 2 of secondary  ⬜ Form 3 of secondary  ⬜ Form 4 of secondary  ⬜ Post-secondary, tertiary and higher education  ⬜ Other (specify) ______________ |
| **2.5** | What is the highest level of education you have attended? (for Nigeria) | 0 No education or pre-school/nursery school only  1 Adult literacy education  2 Quranic school  3 Primary 1  4 Primary 2  5 Primary 3  6 Primary 4  7 Primary 5  8 Primary 6  9 Junior secondary 1  10 Junior secondary 2  11 Junior secondary 3  12 Senior secondary 1  13 Senior secondary 2  14 Senior secondary 3  15 Post-secondary, tertiary and higher education  16 Other (specify) ______________ |
| **2.5** | What is the highest level of education you have attended? (for Zimbabwe) | ⬜ No education or pre-school/nursery school only  ⬜ Adult literacy education  ⬜ Grade 1 of primary education  ⬜ Grade 2 of primary education  ⬜ Grade 3 of primary education  ⬜ Grade 4 of primary education  ⬜ Grade 5 of primary education  ⬜ Grade 6 of primary education  ⬜ Grade 7 of primary education  ⬜ Form 1  ⬜ Form 2  ⬜ Form 3  ⬜ Form 4  ⬜ Form 5  ⬜ Form 6  ⬜ Post-secondary, tertiary and higher education  ⬜ Other (specify) ______________ |
| **2.6** | What is your current employment status (*Allow for selection of multiple answers*) | ⬜ Working on household farming, livestock or fishing activities  ⬜ Running or helping in a non-farm business for yourself or the household (for example as a trader, shop‐keeper, barber, dressmaker, carpenter or taxi driver)  ⬜ Working in formal employment for a wage, salary, commission, or any payment in kind, including doing paid apprenticeship, domestic work or paid farm work, excluding short-term labour  ⬜ Working in casual part-time, short-term labor, depending on the availability of work  ⬜ Student  ⬜ Housekeeper  ⬜ Unemployed ***(go directly to question 2.9)***  ⬜ Other: Specify ___________ |
| **2.7** | Do you receive a regular salary? This means money that is paid by the employer daily, weekly, or monthly | ⬜ Yes  ⬜ No ***(go directly to question 2.9)*** |
| **2.8** | How much is your regular salary? | Amount:____, ___. per month in _______ ***(indicate the currency)***  ***If amount given for another frequency (e.g., daily or yearly), adjust to per month above and provide original amount given by respondent:***  *Amount: _________ per ______ (specify daily, weekly, monthly, yearly or any other frequency)* |
| **2.9** | Are you involved in growing food or raising farm animals for yourself or your family? | ⬜ Yes  ⬜ No ***(go directly to question 2.11)*** |
| **2.10** | What is the approximate value of the food you have grown or amount you have made from animal raising in the past year? (*Ask for past season and then past month if they struggle to answer for a year*) | Past year: Amount: ____, ___ in _______ ***(indicate the currency)***  Past season: Amount: ____, ___ in _______ ***(indicate the currency)***  Past month: Amount: ____, ___ in _______ ***(indicate the currency)*** |
| **2.11** | Do you receive any other income from your labour (monetary or in-kind i.e., goods or services given to you in return for your labour) | ⬜ Yes  ⬜ No ***(go directly to question 2.13)*** |
| **2.12** | What is the approximate level of other income that you receive from your labour | Amount: ____, ___per month in _______ ***(indicate the currency)***  *If amount given for another frequency (e.g., daily or yearly), adjust to per month above and provide original amount given by respondent:*  *Amount: _________ per ________ (specify daily, yearly or any other frequency)* |
| **2.13** | What is the approximate total income of your household, per month? | Amount: ____, ___ in _______ ***(indicate the currency)*** |
| **2.14** | In the past week, have you been worried that your household would not have enough food? | ⬜ Yes  ⬜ No |
| **2.15** | 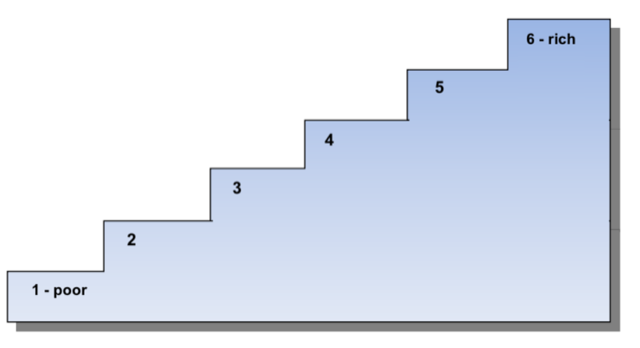 Imagine six steps, where on the bottom, the first step, stand the poorest people, and on the highest step, the sixth, stand the rich. SHOW THE PICTURE OF THE STEPS. On which step are you today? | ⬜ 1  ⬜ 2  ⬜ 3  ⬜ 4  ⬜ 5  ⬜ 6 |

| **3. HEALTH STATUS AND COSTS PRIOR TO THIS VISIT** | | |
| --- | --- | --- |
| **3.1** | Have you experienced any of the following symptoms in the last 2 weeks? ***(Check all that apply)*** | ⬜ Fever or chills  ⬜ Cough  ⬜ Shortness of breath or difficulty breathing  ⬜ Fatigue, tiredness  ⬜ Muscle or body aches and pains  ⬜ Headache  ⬜ New loss of taste or smell  ⬜ Sore throat  ⬜ Chest pain or pressure  ⬜ Congestion or runny nose  ⬜ Nausea or vomiting  ⬜ Diarrhea  ⬜ Red/pink eye or watery eye  ⬜ Other symptoms (specify) __________________  ⬜ No symptom but contact of a person who has tested positive for COVID ***(if no symptom, go directly to question 3.8)*** |
| **3.2** | On what date did the first of the symptoms you just described start? | **\| d \| d \| \| m \| m \| m \| \| y \| y \| y \| y \|** |
| **3.3** | Since the symptoms started, have you had to stop or reduce your normal activities (including activities producing food within the household) because of this/these symptom(s)? | ⬜ Yes  ⬜ No ***(if no, go directly to question 3.7)*** |
| **3.4** | How many days of leave from work (including time producing food within the household) have you taken because of this/these symptom(s)? | _________ days ***(indicate 0 if none)*** |
| **3.5** | On the days you worked, by how many hours per day have you had to reduce your work (including time producing food within the household) because of this/these symptom(s)? | _________ hours per day worked ***(indicate 0 if none)*** |
| **3.6** | How much income (including salaried and non-salaried income) have you lost because of these symptoms? | Amount: ____, ___ in _______ ***(indicate the currency)*** |
| **3.7** | For how many days and how many hours per day did anyone else in the household have to stop working to care for you in relation with these symptoms? | _________ hours per day for _________ days ***(indicate 0 if nobody had to stop working to care for the respondent)*** |
| **3.8** | Have you isolated or taken any precaution to avoid transmitting COVID before testing today? | ⬜ Isolated  ⬜ Wore a mask  ⬜ Other precaution(s) (specify) ______  ⬜ No precaution |
| **3.9** | Have you sought testing and/or care because of these symptoms prior to coming to this site? How many times? | List the different times the respondent sought testing or care seeking and the type of visit below:  ⬜ No testing or care seeking ***(if no, go directly to question 3.9)***  ⬜ Yes, sought testing and/or care **(specify below)** |
| **3.9b** | If yes, specify number and types of visits e.g., self-testing, tested at health centre, doctor/traditional doctor consultation, bought drugs, etc. | Testing and/or care-seeking 1: _______ ***(indicate type of testing or care sought)*** on **\| d \| d \| \| m \| m \| m \| \| y \| y \| y \| y \|**  Testing and/or care-seeking 2: _______ ***(indicate type of testing or care sought)*** on **\| d \| d \| \| m \| m \| m \| \| y \| y \| y \| y \|**  Testing and/or care-seeking 3: _______ ***(indicate type of testing or care sought)*** on **\| d \| d \| \| m \| m \| m \| \| y \| y \| y \| y \|**  Testing and/or care-seeking 4: _______ ***(indicate type of testing or care sought)*** on **\| d \| d \| \| m \| m \| m \| \| y \| y \| y \| y \|**  Testing and/or care-seeking 5: _______***(indicate type of testing or care sought)*** on **\| d \| d \| \| m \| m \| m \| \| y \| y \| y \| y \|**  ***(if no testing or care, go directly to question 3.11)*** |
| **3.10** | How much money did you have to pay for this testing and/or care, including direct service payment and transport costs? Indicate amounts per time sought testing and/or care as above. | Amount for testing and/or care seeking 1: ____, ___ in _______ ***(indicate the currency)***  Amount for testing and/or care seeking 2: ____, ___ in _______ ***(indicate the currency)***  Amount for testing and/or care seeking 3: ____, ___ in _______ ***(indicate the currency)***  Amount for testing and/or care seeking 4: ____, ___ in _______ ***(indicate the currency)***  Amount for testing and/or care seeking 5: ____, ___ in _______ ***(indicate the currency)*** |
| **3.11** | How much time did you spend specifically to seek that testing and/or care (including time at the site and travel time from where you were before to where you were going to after receiving care) Indicate duration spent per time sought testing and/or care. | Time spent for testing and/or care seeking 1: ______ hours ________ minutes  Time spent for testing and/or care seeking 2: ______ hours ________ minutes  Time spent for testing and/or care seeking 3: ______ hours ________ minutes  Time spent for testing and/or care seeking 4: ______ hours ________ minutes  Time spent for testing and/or care seeking 5: ______ hours ________ minutes |
| **3.12** | How much money did you spend on any other expense, for example to buy extra food or supplies or for accommodation related to your symptoms? | Amount for testing and/or care seeking 1: ____, ___ in _______ ***(indicate the currency)***  Amount for testing and/or care seeking 2: ____, ___ in _______ ***(indicate the currency)***  Amount for testing and/or care seeking 3: ____, ___ in _______ ***(indicate the currency)***  Amount for testing and/or care seeking 4: ____, ___ in _______ ***(indicate the currency)***  Amount for testing and/or care seeking 5: ____, ___ in _______ ***(indicate the currency)*** |

*I am now going to ask you questions about your current visit*

| **4. QUESTIONS AROUND THE CURRENT VISIT** | | |
| --- | --- | --- |
| **4.1** | What health service(s) did you seek today? | ⬜ COVID-19 testing  ⬜ COVID vaccination  ⬜ COVID prescription  ⬜ Other COVID-related service (specify) ________  ⬜ Non-COVID consultation  ⬜ Non-COVID vaccination  ⬜ Non-COVID prescription  ⬜ Other non-COVID-related service (specify) _______  ⬜ Other (specify): ______________________ |
| **4.2** | What other health services did you access today in addition to COVID-19 testing? | ⬜ COVID vaccination  ⬜ COVID prescription  ⬜ Other COVID-related service (specify) ________  ⬜ Non-COVID consultation  ⬜ Non-COVID vaccination  ⬜ Non-COVID prescription  ⬜ Other non-COVID-related service (specify) _______  ⬜ Other (specify): ______________________ |
| **4.3** | Did you have to travel to this testing site today specifically to get the health services you accessed today? *We want to understand if you undertook any additional travel to receive the health services you sought today as compared to what you would have done otherwise* | ⬜ Yes  ⬜ No ***(if no, go directly to question 4.9)*** |
| **4.4** | What mode of transport did you use to get to this testing location from the place you were at before? | ⬜ Walked  ⬜ Took bicycle  ⬜ Took public transport  ⬜ Used own car or motorcycle  ⬜ Took taxi  ⬜ Other (specify): ______________________ |
| **4.5** | How long did it take you to get to the testing site from the place you were at before? | _______ hours _______ minutes |
| **4.6** | How much transport money did you spend to get to this testing site today from the place you were at before? | Amount: ____, ___ in _______ ***(indicate the currency,***  ***( write 0 if nothing spent)*** |
| **4.7** | How long do you anticipate that your trip to get to the place you are going next will take? | _______ hours _______ minutes |
| **4.8** | How much transport money will you spend on your trip to get to the place you are going to next? | Amount: ____, ___ in _______ ***(indicate the currency,***  ***( write 0 if nothing spent)*** |
| **4.9** | At what time did you arrive at the testing site today? | **\| h \| h \|:\| m \| m \| *[Based on a 24 hour clock eg.15:30]*** |
| **4.10** | How much time in minutes did you spend waiting at the testing site today before receiving the first of the services you came for? | _______ hours _______ minutes ***(indicate 0 if no wait time)*** |
| **4.11** | How much time did you spend getting your COVID-19 test today? This includes time spent seeing a demonstration of how to do the test (if any) and time spent testing | _______ minutes |
| **4.12** | How much time did you spend waiting for the results of the test? | _______ minutes ***(if no, go directly to question 4.12)*** |
| **4.13** | How much time did you spend on post-test follow-up (this may include receiving advice, being asked about your contacts, receiving drugs or tools such as masks) | _______ minutes ***(indicate 0 if no follow-up time)*** |
| **4.14** | Did you have to pay any fees to take the COVID-19 test? This includes consultation, registration, the test kit, and health passport. If so, how much? | ⬜ Yes, amount Amount: ____, ___ in _______ ***(indicate the currency)***  ⬜ No |
| **4.15** | How much money have you paid for each of the other health services you received at this site today (direct service payment)? | Amount: ____, ___ in _______ ***(indicate the currency)*** |
| **4.16** | How much additional money aside from what you stated above did you spend on your visit today, for example to buy extra food or supplies or for accommodation? | Amount: ____, ___ in _______ ***(indicate the currency)*** |
| **4.17** | Did you need to take time off work for your visit to the testing site today? | ⬜ Yes  ⬜ No (***if no time off taken, go directly to question 4.17)*** |
| **4.18** | How much money would you have earned (from salaried job or any other work you do) during the time you took off to get tested for COVID-19 today? | Amount: ____, ___ in _______ ***(indicate the currency)*** |
| **4.19** | Did you have to pay for anyone to cover your regular duties while getting the COVID-19 test? This includes to take care of your children, supervise your shop or perform your agricultural activities? | ⬜ Yes  ⬜ No (***if no time off taken, go directly to question 4.19)*** |
| **4.20** | If yes how much did you pay for someone to cover your regular duties? | Amount: ____, ___ in _______ ***(indicate the currency)*** |
| **4.21** | How many people came to this testing site to accompany you? Exclude people who came to the site with you to receive health services for themselves | Number of people: ____ ***(if nobody accompanied the respondent, indicate 0 and skip to question 4.22)*** |
| **4.22** | What is their occupation? ***(Fill for as many people as applies)*** | Person 1: ______  Person 2: ______  Person 3: ______  Person 4: ______  Person 5: ______  Person 6: ______ |
| **4.23** | Did they need to take time off work to accompany you for your visit to the testing site today?  ***If more than 1 person accompanying the respondent, ask for each person listed in 4.20***: *“For person X whose occupation is _____, did they have to take time off work to accompany you?”* | Person 1: ⬜ Yes ⬜ No  Person 2: ⬜ Yes ⬜ No  Person 3: ⬜ Yes ⬜ No  Person 4: ⬜ Yes ⬜ No  Person 5: ⬜ Yes ⬜ No  Person 6: ⬜ Yes ⬜ No |
| **4.24** | Did you do the test yourself or did somebody else do it for you? | ⬜ I did the test myself *(this includes if you received guidance on how to take the test)*  ⬜ A health professional did the test for me |
| **4.25** | What type of test did you get? | ⬜ Antigen test  ⬜ PCR test  ⬜ Not sure |
| **4.26** | What is your COVID-19 status? | ⬜ Negative ⬜ Positive ⬜ Prefer not to say ⬜ I don’t know ***(if no, skip 4.25)*** |
| **4.27** | What did you receive following the diagnosis e.g., advice, drugs, referral, masks, testing kits, etc? | ⬜ Advice ____________  ⬜ Masks (specify e.g., surgical) ________  ⬜ Monitoring tools (specify e.g., pulse oximeter) _______  ⬜ Antivirals (specify name if known) _______  ⬜ Other treatment (specify name if known) _______  ⬜ Drug prescription (specify name if known) _______  ⬜ Referral (specify) ____________________________  ⬜ Financial or in-kind support  ⬜ Kits for contacts (specify) __________  ⬜ Other (specify) ______________________ |
