# Supplementary material for: Economic barriers to diagnostic equity: A multi-country analysis of patient costs for rapid SARS-CoV-2 testing in sub-Saharan Africa
Source: PLoS One. 2026 Jun 2;21(6):e0350288. doi: 10.1371/journal.pone.0350288 (PMC13229357; doi:10.1371/journal.pone.0350288)
Supplement: Attachment — Submitted filename: Point by Point Response_GB.docx [file pone.0350288.s004.docx]

**POINT BY POINT RESPONSE:**

PONE-D-26-08248. Economic barriers to diagnostic equity: a multi-country analysis of patient costs for rapid SARS-CoV-2 testing in sub-Saharan Africa

**ACADEMIC EDITOR:**

1. please add the following details:

a - Financial disclosure (funding details) – please add grant number

**Answer: We have added Unitaid grant number (2017-16-PSI-STAR to KH). See cover letter.**

b - Ethical approval – add approvals number

**Answer: We have added ethical approval numbers. See ethics considerations (pag3 8 – 9).**

Please submit your revised manuscript by May 24 2026 11:59PM. If you will need more time than this to complete your revisions, please reply to this message or contact the journal office at [plosone@plos.org](mailto:plosone@plos.org). When you're ready to submit your revision, log on to https://www.editorialmanager.com/pone/ and select the 'Submissions Needing Revision' folder to locate your manuscript file.

Please include the following items when submitting your revised manuscript:

- A letter that responds to each point raised by the academic editor and reviewer(s). You should upload this letter as a separate file labeled 'Response to Reviewers'.

**Answer: We uploaded a separate file labeled response to reviewers.**

- A marked-up copy of your manuscript that highlights changes made to the original version. You should upload this as a separate file labeled 'Revised Manuscript with Track Changes'.

**Answer: We uploaded a separate file labeled “Revised manuscript with track changes”.**

- An unmarked version of your revised paper without tracked changes. You should upload this as a separate file labeled 'Manuscript'.

**Answer: We uploaded another unmarked version of the revised manuscript and labelled ‘Manuscript’**

If you would like to make changes to your financial disclosure, please include your updated statement in your cover letter. Guidelines for resubmitting your figure files are available below the reviewer comments at the end of this letter.

**Answer: Not applicable.**

When submitting your revision, we need you to address these additional requirements.

1. Please ensure that your manuscript meets PLOS ONE's style requirements, including those for file naming. The PLOS ONE style templates can be found at 
<https://journals.plos.org/plosone/s/file?id=wjVg/PLOSOne_formatting_sample_main_body.pdf> and 
<https://journals.plos.org/plosone/s/file?id=ba62/PLOSOne_formatting_sample_title_authors_affiliations.pdf>

**Answer: Yes, the manuscript meets PLOS ONE’s style requirements.**

2. Please include a complete copy of PLOS’ questionnaire on inclusivity in global research in your revised manuscript. Our policy for research in this area aims to improve transparency in the reporting of research performed outside of researchers’ own country or community. The policy applies to researchers who have travelled to a different country to conduct research, research with Indigenous populations or their lands, and research on cultural artefacts. The questionnaire can also be requested at the journal’s discretion for any other submissions, even if these conditions are not met. Please find more information on the policy and a link to download a blank copy of the questionnaire here: <https://journals.plos.org/plosone/s/best-practices-in-research-reporting>. Please upload a completed version of your questionnaire as Supporting Information when you resubmit your manuscript.

**Answer: Yes, we have filled the questionnaire and uploaded it as S2.**

3. Thank you for stating the following in the Acknowledgments Section of your manuscript:

“This study was supported by UNITAID through Population Services International (PSI). The funders were not involved in the study design, data collection or analysis, the decision to publish, or manuscript preparation. The authors are solely responsible for the content, which does not necessarily reflect the official positions of UNITAID or PSI.”

We note that you have provided funding information that is not currently declared in your Funding Statement. However, funding information should not appear in the Acknowledgments section or other areas of your manuscript. We will only publish funding information present in the Funding Statement section of the online submission form.

Please remove any funding-related text from the manuscript and let us know how you would like to update your Funding Statement. Currently, your Funding Statement reads as follows:

“This study was supported by UNITAID through Population Services International (PSI). The funders were not involved in the study design, data collection or analysis, the decision to publish, or manuscript preparation. The authors are solely responsible for the content, which does not necessarily reflect the official positions of UNITAID or PSI.”

Please include your amended statements within your cover letter; we will change the online submission form on your behalf.

**Answer: We have removed funding statement from the acknowledgement section. In our cover letter you will find the amended funding statement as follows: “This study was supported by UNITAID grant (2017-16-PSI-STAR to KH) through Population Services International (PSI). The funders were not involved in the study design, data collection or analysis, the decision to publish, or manuscript preparation. The authors are solely responsible for the content, which does not necessarily reflect the official positions of UNITAID or PSI.”**

4. PLOS requires an ORCID iD for the corresponding author in Editorial Manager on papers submitted after December 6th, 2016. Please ensure that you have an ORCID iD and that it is validated in Editorial Manager. To do this, go to ‘Update my Information’ (in the upper left-hand corner of the main menu), and click on the Fetch/Validate link next to the ORCID field. This will take you to the ORCID site and allow you to create a new iD or authenticate a pre-existing iD in Editorial Manager.

**Answer: I have added my ORCID iD to my account.**

5. We note that you have referenced “An unpublished study” which has currently not yet been accepted for publication. Please remove this from your References and amend this to state in the body of your manuscript: as detailed online in our guide for authors
<http://journals.plos.org/plosone/s/submission-guidelines#loc-reference-style>

**Answer: The study cited was a manuscript on preprint server with citable DOI, which is acceptable by PLOSOne. Thus we have deled the word “unpublished”.**

6. We note that there is identifying data in the Supporting Information file “Dataset.xlsx”. Due to the inclusion of these potentially identifying data, we have removed this file from your file inventory. Prior to sharing human research participant data, authors should consult with an ethics committee to ensure data are shared in accordance with participant consent and all applicable local laws.

Data sharing should never compromise participant privacy. It is therefore not appropriate to publicly share personally identifiable data on human research participants. The following are examples of data that should not be shared:

-Name, initials, physical address
-Ages more specific than whole numbers
-Internet protocol (IP) address
-Specific dates (birth dates, death dates, examination dates, etc.)
-Contact information such as phone number or email address
-Location data
-ID numbers that seem specific (long numbers, include initials, titled “Hospital ID”) rather than random (small numbers in numerical order)

Data that are not directly identifying may also be inappropriate to share, as in combination they can become identifying. For example, data collected from a small group of participants, vulnerable populations, or private groups should not be shared if they involve indirect identifiers (such as sex, ethnicity, location, etc.) that may risk the identification of study participants.

Additional guidance on preparing raw data for publication can be found in our Data Policy (<https://journals.plos.org/plosone/s/data-availability#loc-human-research-participant-data-and-other-sensitive-data>) and in the following article: <http://www.bmj.com/content/340/bmj.c181.long>.

Please remove or anonymize all personal information “Dates and Patient ID”, ensure that the data shared are in accordance with participant consent, and re-upload a fully anonymized data set. Please note that spreadsheet columns with personal information must be removed and not hidden as all hidden columns will appear in the published file.

**Answer: Thank you. We have ensured that we have anonymized all personal information in the manuscript and other documents uploaded.**

7. Please include captions for your Supporting Information files at the end of your manuscript, and update any in-text citations to match accordingly. Please see our Supporting Information guidelines for more information: [http://journals.plos.org/plosone/s/supporting-information.](http://journals.plos.org/plosone/s/supporting-information.%E2%80%8B)

**Answer. We have included captions in the two supporting information files.**

8. If the reviewer comments include a recommendation to cite specific previously published works, please review and evaluate these publications to determine whether they are relevant and should be cited. There is no requirement to cite these works unless the editor has indicated otherwise.

**Answer: Noted**

9. Please review your reference list to ensure that it is complete and correct. If you have cited papers that have been retracted, please include the rationale for doing so in the manuscript text, or remove these references and replace them with relevant current references. Any changes to the reference list should be mentioned in the rebuttal letter that accompanies your revised manuscript. If you need to cite a retracted article, indicate the article’s retracted status in the References list and also include a citation and full reference for the retraction notice.

**Answer: We have reviewed the reference list and ensured that it is complete and correct.**

**REVIEWER #1:**

The manuscript was well written with data and results well presented. The authors explained well why the study is important in planning for future outbreaks and were able to show what was obtained in three African countries. There may be need for a much broader study involving more countries so as to have a good picture of what was obtained in sub-Saharan Africa and solutions proffered.

**Response: Thank you for this comment. We appreciate the reviewer’s recognition of the importance of our multi-country analysis and its relevance for informing future outbreak preparedness. We have now strengthened the manuscript to explicitly acknowledge this limitation and to highlight the need for broader, regionally representative studies. We also emphasize that our findings provide a useful foundation for future large-scale, multi-country research aimed at informing diagnostic policy across sub-Saharan Africa. These revisions have been incorporated into the Discussion and Limitations sections of the manuscript as shown below:**

**Discussion:**

***“While this study provides important insights across three sub-Saharan African countries, future research involving a larger number of countries would be valuable to generate a more comprehensive regional picture of patient-level costs and affordability of diagnostic testing. Such broader analyses could further inform context-specific and region-wide strategies to improve equitable access to diagnostics.”***

**Limitation paragraph in Discussion:**

***“This study presents a number of limitations. It utilized a limited number of test settings and was conducted in only three countries, which may limit the generalizability of findings across the broader sub-Saharan African region. While Nigeria, Malawi, and Zimbabwe were selected to reflect diverse implementation contexts, a larger multi-country study would provide a more comprehensive understanding of patient costs and affordability across different health systems in the region.”***

**REVIEWER #2:**

This manuscript addresses an important and underexplored aspect of diagnostic access in sub-Saharan Africa, namely the patient-side economic burden of SARS-CoV-2 rapid diagnostic testing. The multi-country design and focus on affordability provide valuable insights for health policy and future pandemic preparedness. Overall, the study is timely, relevant, and contributes meaningfully to the literature.

The manuscript is generally well-structured, and the conclusions are broadly supported by the data. However, several areas require clarification and minor strengthening to improve transparency, rigor, and interpretability.
Review comments
1. Clarity of costing methodology
While the study outlines the components of patient costs (transport, medical, non-medical, productivity loss), further clarification is needed on:
• How productivity loss was calculated in practice, particularly the substitution of missing self-reported income with minimum wage estimates
• Whether sensitivity analyses were conducted to assess the robustness of these assumptions
• How extreme values (e.g., very high SD in Nigeria costs) were handled

Given that productivity loss is a major cost driver, more transparency here is essential.

**Response: Thank you for this insightful comment on the clarity of our costing methodology. We have revised the manuscript to provide a more detailed description of how productivity loss was calculated. Specifically, productivity loss was estimated based on the total time spent accessing testing (including waiting time, testing time, and time to receive results), which was converted into hours. This time was then monetized using either self-reported income loss (where available) or a standardized estimate based on the national minimum wage to account for accompanying persons or missing data. This approach ensured consistency and allowed us to capture opportunity costs even when self-reported income data were incomplete (See page 6).**

**Regarding sensitivity analyses, we acknowledge their importance; however, given the descriptive nature of our study and reliance on observed field data, we did not conduct formal sensitivity analyses. This has now been explicitly stated as a limitation (see page 19).**

**Finally, we clarified that all observed values, including extreme values contributing to large standard deviations (e.g., in Nigeria), were retained to reflect real-world variability in patient costs rather than being truncated or excluded (see page 7).**

**These clarifications have been incorporated into the Methods and Limitations sections of the manuscript.**

2. Statistical reporting and rigor
The analysis is largely descriptive, which is acceptable given the study objectives. However:
• No confidence intervals or uncertainty measures are presented for key estimates
• There is no indication of statistical comparison across countries or modalities
• The rationale for not performing comparative statistical tests should be stated
Including basic inferential comparisons or explicitly justifying their absence would strengthen the analytical rigor.

**Response: Thank you for this valuable comment. We agree that presenting measures of uncertainty strengthens the interpretation of descriptive findings. In response, we have now included 95% confidence intervals for key cost estimates (cost per client) across countries, calculated using the reported means, standard deviations, and sample sizes (See page 11).**

**Regarding statistical comparisons across countries and testing modalities, our study was designed as a descriptive, multi-country implementation analysis rather than a hypothesis-testing study. Differences in sampling strategies, contextual factors, and heterogeneity in testing modalities across countries limit the validity of direct statistical comparisons. We have now clarified this rationale in the Method section (page 6).**

3. Interpretation of catastrophic health expenditure
The use of a 10% threshold is standard, but:
• The manuscript should acknowledge that this threshold may not fully capture financial hardship across heterogeneous income settings
• There should be a brief discussion of how income measurement (self-reported) may introduce bias

**Response: Thank you for this important comment regarding the interpretation of catastrophic health expenditure.**

**We agree that while the 10% threshold is widely used in the literature, it may not fully capture the extent of financial hardship across heterogeneous income settings. We have revised the manuscript to acknowledge this limitation and to emphasize that the threshold should be interpreted cautiously, particularly in low-income populations where even smaller expenditures may constitute a significant burden (See page 15 -16).**

**We have also added clarification regarding the use of self-reported income data, noting the potential for reporting bias and its implications for estimating catastrophic health expenditure (see page 20).**

4. Generalizability
The limitations section correctly notes selection bias (only those who sought testing were included), but this point should be strengthened:
• The study likely underestimates true costs, especially for those unable to access care
• This limitation has important implications for interpreting affordability estimates

**Response: Thank you. We agree that restricting the sample to individuals who successfully accessed testing may lead to an underestimation of the true economic burden, particularly for individuals who were unable to seek care due to financial or access barriers. We have strengthened the limitations section to explicitly acknowledge this potential selection bias and its implications for interpreting both cost and affordability estimates. Specifically, we now emphasize that our findings likely underestimate the full economic burden of testing and that affordability estimates, including catastrophic health expenditure, should be interpreted as conservative. These revisions have been incorporated into the limitations section of the manuscript (See Page 19).**

Other Comments
1. Typographical and formatting issues
• Minor inconsistencies (spacing, punctuation, duplicated percentage symbols in Table 2)
• Ensure uniform reporting of currency (US$ vs $)

**Response: We have corrected minor typographical and formatting inconsistencies throughout the manuscript, including spacing, punctuation, and duplicated percentage symbols (e.g., in Table 2). We have also standardized the reporting of currency to “US$” across the entire manuscript to ensure consistency.**

2. Terminology consistency
• “Drug stores” vs “community pharmacies” should be clearly defined early and used consistently

**Response: We agree that consistent terminology is important for clarity. We have now clearly defined “community pharmacies” and “drug stores” in the Methods section and ensured that these terms are used consistently throughout the manuscript (see page 5 to 6).**

3. Results clarity
• The very high standard deviation in Nigeria (SD = 16.0) should be briefly explained in the text

**Response: We have added a brief explanation in the Results section to clarify the high standard deviation observed in Nigeria (SD = 16.0). Specifically, we note that this reflects substantial variability in patient-incurred costs, particularly driven by differences in travel costs and productivity losses (see 11).**

4. Figure and tables
• Figure 1 (supplementary files)could benefit from clearer labeling (x-axis titles and formatting)
• Some tables are dense and may need slight simplification for readability especially table 2.

**Response: We have improved the labeling and formatting of Figure 1, including clearer axis titles and presentation, to enhance interpretability. We have revised Table 2 to improve readability by removing repeated denominators, standardizing the presentation of values as counts with percentages, and organizing variables into clear sections. We also corrected formatting inconsistencies and minor typographical errors (See pages 9 – 11).**
